# Applicability of Cost-Effective GNSS Sensors for Crustal Deformation Studies

**DOI:** 10.3390/s22010350

**Published:** 2022-01-04

**Authors:** Lavinia Tunini, David Zuliani, Andrea Magrin

**Affiliations:** National Institute of Oceanography and Applied Geophysics (OGS), 33100 Udine, Italy; dzuliani@inogs.it (D.Z.); amagrin@inogs.it (A.M.)

**Keywords:** GNSS, low-cost receivers, cost-effective sensors, crustal deformation, GAMIT-GLOBK

## Abstract

The geodetic monitoring of the continuous crustal deformation in a particular region has traditionally been the prerogative of the scientific communities capable of affording high-price geodetic-class instruments to track the tiny movements of tectonic plates without losing precision. However, GNSS technology has been continuously and rapidly growing, and in the last years, new cost-efficient instruments have entered the mass market, gaining the attention of the scientific community for potentially being high-performing alternative solutions. In this study, we match in parallel a dual-frequency low-cost receiver with two high-price geodetic instruments, all connected to the same geodetic antenna. We select North-East Italy as testing area, and we process the data together with the observations coming from a network of GNSS permanent stations operating in this region. We show that mm-order precision can be achieved by cost-effective GNSS receivers, while the results in terms of time series are largely comparable to those obtained using high-price geodetic receivers.

## 1. Introduction

The Global Navigation Satellite Systems (GNSS) provide a globally extended data set of primordial importance for a wide range of applications, from the crustal deformation analysis to the near-surface processes monitoring (i.e., landslide, bridge and dams damages, ice sheet movements, etc.) and surveying. Geodetic-class (i.e., high-cost) instruments allow accuracies in the order of millimeters. However, high precision usually implies high cost, and this represents a strong limitation not only for the institutions and scientific communities with a restricted budget but also for those carrying out monitoring projects where there is a higher risk of instrument damage [1,2,3].

In recent years, cost-effective sensors have entered the mass market, progressively gaining the interest of the scientific community as alternative solutions for a variety of applications both in static and kinematic modes. Biagi et al. [4] investigated the performance of single-frequency low-cost receivers for local monitoring in kinematic mode, detecting 15 mm of horizontal displacement using a geodetic-class receiver as the base station. Caldera et al. [5] analyzed the performance of u-blox EVK-6T receivers in open-sky favorable conditions, showing that movements of 2–3 mm can be detected when a short baseline with daily solutions is used. Recent tests, in both static and kinematic mode, published by Hamza et al. [6], suggest that low-cost GNSS instruments can detect displacements from 10 mm upwards with a high level of reliability, although such instruments perform slightly worse as far as accuracy is concerned. Some studies pointed out that matching low-cost receivers with geodetic-class antennas can be a successful strategy for surveying or real-time application purposes. Cina and Piras [7] show that the accuracy achieved in post-processing with a mass-market single-frequency receiver allows reaching mm-order precision especially if combined with a geodetic-class antenna. Similar results were observed by Tsakiri et al. [8], who use low-cost receivers, as the u-blox LEA-6T and NEO-7P, combined with geodetic-class antennas, reaching the accuracy levels of 1–2 cm (at 95% confidence level) required for surveying applications such as land development, digital mapping, hydrography, etc. More recently, Poluzzi et al. [9] analyzed the capability of a system formed by low-cost receivers and high-cost antennas for monitoring slow displacements or for obtaining suitable real-time solutions for early warning aims. Their results indicate that, considering daily observations, the system can reach precisions of less than 1 mm RMS for horizontal components and 1–1.5 mm RMS for the vertical one, while real-time solutions RMS range from 4 to 8 mm for horizontal and vertical components, respectively. Garrido-Carretero et al. [10] confirm the validity of positioning performance of low-cost receivers (namely u-blox NEO-M8P) over short baselines, for real-time positioning, with an uncertainty of ±5.5 mm for the horizontal components and ±11 mm for vertical one.

Our study aims to prove that cost-effective double frequency receivers combined with geodetic-class antennas can provide reliable results not only for real-time applications, but also for crustal deformation purposes. Generally, for continuous deformation studies, scientists have primarily relied on high-price geodetic receivers capable of tracking the tiny relative movements of tectonic plates without losing precision. However, the entrance of high-performing cost-effective geodetic instrumentation in the market makes us wonder whether such instrumentation guarantees reliable results also for regional deformation studies. To answer this, we installed in parallel a dual-frequency low-cost receiver (u-blox ZED F9P) and two high-cost ones, all connected to the same geodetic-class antenna. By using two geodetic-class receivers, we can evaluate the variability of results for the same instrument class and compare it with the results of low-cost one. We process the data of such a system together with the data coming from a GNSS network active in North-East Italy; hence, we compare the time-series obtained using low-cost geodetic equipment with those obtained using geodetic-class instruments. Finally, the results are discussed in terms of the reliability of the time-series for depicting the long-term tectonic signal existing in North-East Italy.

## 2. Data Acquisition and Processing

### 2.1. Instrumentation

**Figure 1:**
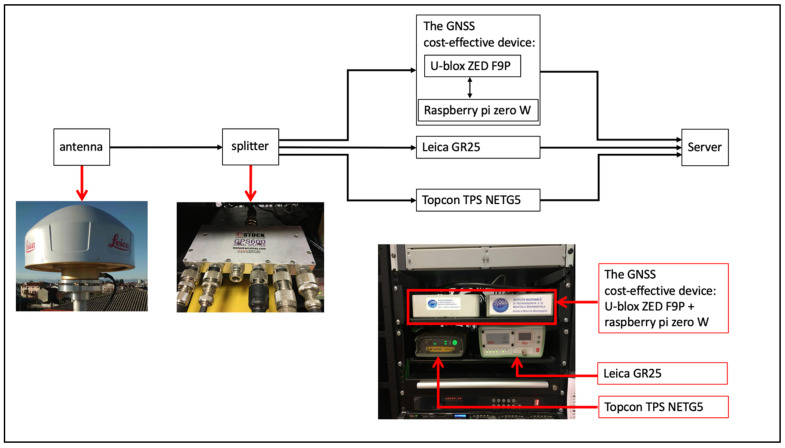
Block diagram of the sensors system. The GNSS cost-effective device is detailed in Figure 2.

**Figure 2:**
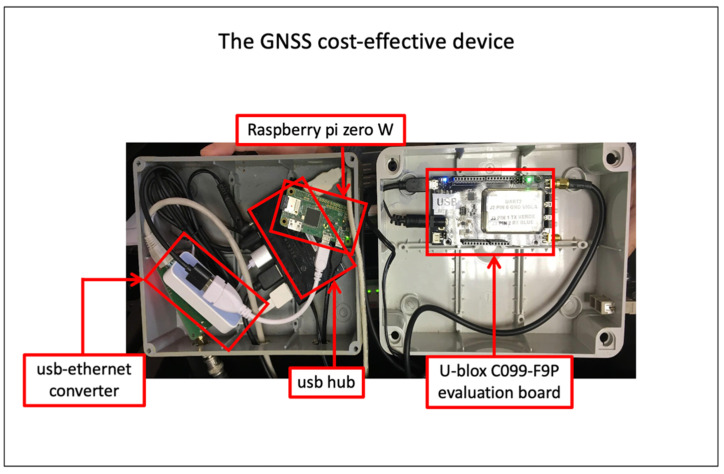
Details of the GNSS cost-effective device shown in Figure 1. The right box includes the u-blox C099-F9P evaluation board (the board includes the u-blox ZED F9P receiver). The left box includes a raspberry pi zero W plugged to a usb hub. The usb hub connects the raspberry pi zero W to the u-blox C099-F9P evaluation board and to the usb-ethernet converter. The latter allows us to access the raspberry pi zero W from the server. All the data tracked by the evaluation board are recorded inside the raspberry pi storage system.

We perform an experiment using a set of three GNSS types of equipment, which we call “Multistation System” (MS). The GNSS equipment of the MS (Figure 1 and Figure 2) is composed of one geodetic-class antenna Leica LEIAR20, matched to three parallelly connected receivers: (i) a GNSS cost-effective device, formed by a cost-effective evaluation board (C099-F9P provided by u-blox) mounting a dual-frequency u-blox ZED-F9P chipset (UDZ2); (ii) a Topcon TPS NETG5 (UDT2); and (iii) a Leica GR25 (UDI2). The MS is located in the headquarters of the Seismological Research Center (CRS) of the National Institute of Oceanography and Applied Geophysics—OGS, in Udine (North-East Italy).

The u-blox C099-F9P evaluation board is equipped with a cost-effective (or also low-cost) ZED-F9P chipset and provides an easy-to-use USB interface to access all the F9P potential. We used the USB interface to plug the evaluation board into a Raspberry Pi zero W (https://www.raspberrypi.org/, accessed on 1 December 2021) Single Board Computer. The Pi runs a Linux Operating System Debian distribution. We used a well-known software called RTKLIB [11], but optimized for low-cost GPS receivers, the RTKLIB explorer ver.2.4.3 demo5 b29d. We developed shell scripts and python codes to combine RTKLIB components and automatically record GNSS data coming from ZED-F9P into Receiver Independent Exchange Format (*RINEX*) hourly files. All the *RINEX* files are collected and archived on a computing server where the processing software is used to perform daily time series. All this work is based on developing the LZER0 low-cost device described in Zuliani et al. [12,13]. The ZED-F9P is a powerful GNSS receiver chipset able to track different GNSS constellations (BeiDou, Galileo, GLONASS, GPS/QZSS) and carrier frequencies (L1C/A, L2C, L1OF, L2OF, E1B/C, E5b, B1I, and B2I) at a very reasonable cost (currently the simplest USB board mounting that chip costs roughly EUR 200 and the evaluation board value is around EUR 500) which makes it a very interesting device to be taken into account for different applications, generally carried out using 10 times higher cost devices.

To be thorough, we report in Table 1 the main characteristics of the three GNSS receiver types used in this study. It can be seen that, besides the economic cost, the major differences lie in the type of tracked signal (e.g., the GNSS cost-effective device can only track L2C, instead Leica and Topcon can both track also L2P), in the number of available GNSS channels and in the sampling rate capability (further details can be found at https://kb.unavco.org/kb/article/unavco-resources-gnss-receivers-434.html, accessed on 1 December 2021).

The station UDI2 is part of the Friuli Regional Deformation Network (FReDNet, http://frednet.crs.inogs.it, accessed on 1 December 2021; Zuliani et al. [14]), consisting of 19 continuously operating GNSS stations, whose raw data are collected by CRS, formatted as *RINEX* files, quality-checked, and released through an ftp server. Real-Time Kinematic (RTK) services are supported as well by the system, and all GNSS data and products are included in an agreement to support the Joint Research Unit EPOS-Italy (https://www.epos-eu.org/, accessed on 1 December 2021). FReDNet is part of a bigger monitoring infrastructure belonging to OGS, counting with seismometers and strong motion instruments as well, devoted to the terrestrial monitoring of North-East Italy [15]. FReDNet has been operating since 2002, and long time-series are available for most FReDNet sites, representing reliable information sources for understanding the long-term deformation undergoing in the region.

### 2.2. Data Processing

The goal of this study is to verify whether low-cost geodetic receivers can be considered reliable instruments for continuous deformation studies.

In order to ensure the link between our MS and the NE-Italy regional deformation pattern, we process the data coming from the three stations of the MS, together with the FReDNet sites, and with some globally scattered sites from the European GNSS network (EUREF) and the International GNSS Service (IGS), for a total of 37 geodetic stations.

We collect raw data with a sampling rate equal to 1 s and format them into *RINEX* files sampled at 30 s. We process the data using the GAMIT-GLOBK software package (ver 10.71, [16]) from 9 February to 9 July 2021. The GAMIT module outputs loosely constrained daily solutions, with coordinates and parameter estimates (i.e., Earth orientation, satellite coordinates, tropospheric and ionospheric delays, atmospheric pressure, and ocean and polar tides, etc.) for each station, together with the covariance matrix. In addition, the GLOBK module implements the Kalman filtering to combine the regional loosely constrained daily solutions with the global daily solutions of the IGS network available from the Massachusetts Institute of Technology IGS Data Analysis Center, in order to tie the resulting position/velocity solutions into a consistent reference frame. The results are single daily position values for each station in the international reference frame ITRF08 [17]. The *glorg* program of GLOBK allows, finally, the coordinates in the European reference frame to be obtained by applying the EURA constraints as described in the ITRF2008 plate motion model of Altamimi et al. [17]. More details on the processing procedure can be found in Rossi et al. [18].

The resulting time-series obtained at UDZ2 using the low-cost geodetic equipment, and at UDT2 and UDI2 using geodetic-class instruments, are presented and discussed in the next section.

## 3. Results and Discussion

A first comparison between the performance of the different receivers can be made by analyzing the number of observations captured by each station. The two geodetic-class receivers (UDI2 and UDT2), i.e., the Leica GR25 and the TPS NETG5 receivers, retrieve a higher percentage of observations (above 90%) with respect to the UDZ2, equipped with the u-blox F9P device. UDZ2 retrieves only half of the observations for various days, even though it is capable of retrieving the 60–70% of the observations for most of the days considered in this study.

**Figure 3:**
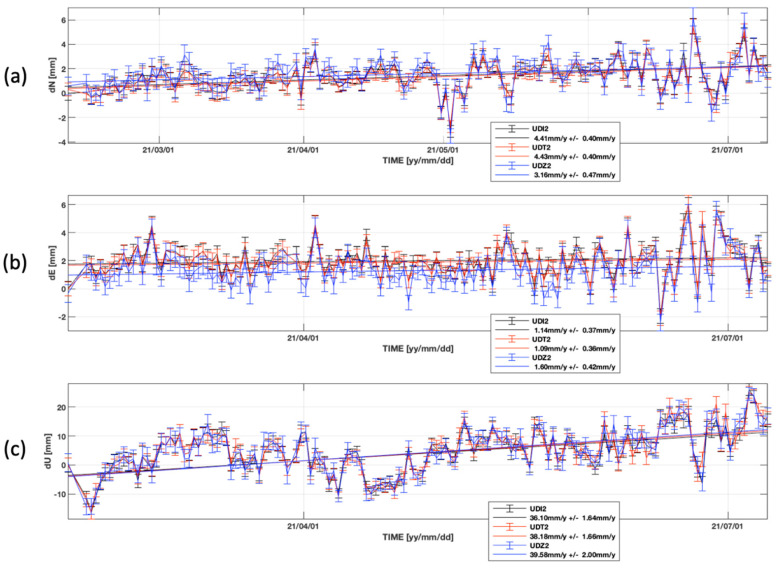
Time-series for MS stations in the considered time interval for the (**a**) North, (**b**) East, and (**c**) Up components of the displacement, with their linear trends overlapped (continuous lines). The starting point is the estimated position of UDI2 calculated using all available data since its installation in mid-2017. The color indicates the station: red and black indicate UDI2 and UDT2, equipped with top-quality Leica and Topcon receivers, respectively; blue represents the UDZ2, equipped with a low-cost GNSS receiver (u-blox). Error bars indicate the dispersion of the daily estimates.

**Figure 4:**
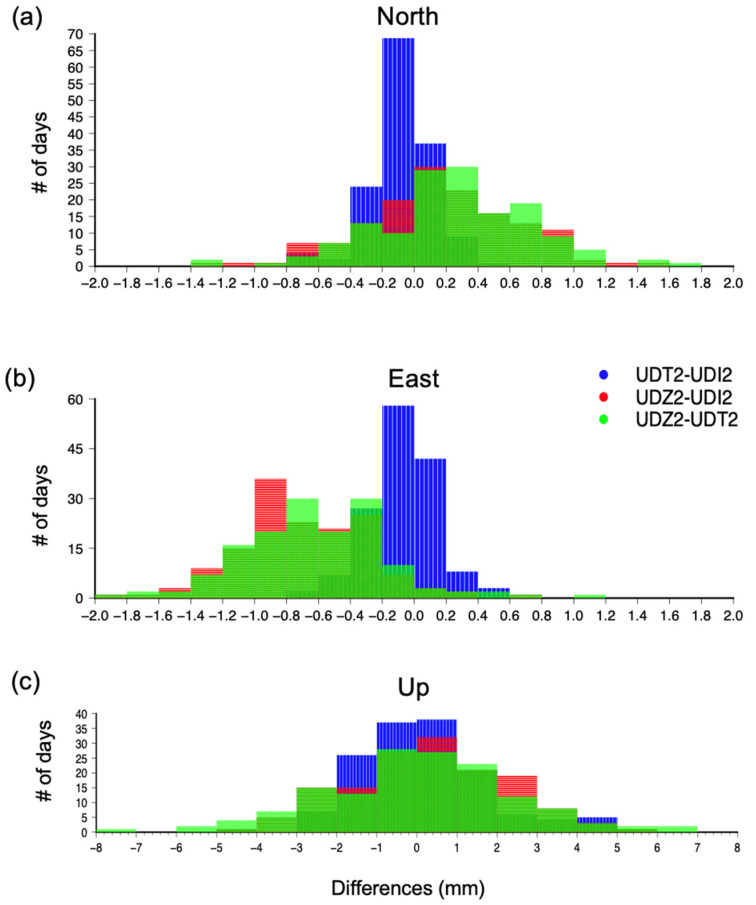
Histograms of the differences between UDT2 and UDI2, UDZ2 and UDI2, UDZ2 and UDT2, for the (**a**) North, (**b**) East, and (**c**) Up displacement components.

Figure 3 shows the time-series of the MS stations for the considered time-interval in the European reference frame starting from the estimated position of UDI2 on 9th February 2021. The trending line of each time series is overlapped. We computed the differences in the daily positions estimated for each station. For each station of the MS and each displacement component, Figure 4 shows the histograms of the differences and Table 2 indicates the maximum differences in the daily estimates, the mean, and the standard deviation.

The results allow us to point out the following:

(i) The similarities in the time series of the MS stations indicate that the daily displacement estimates obtained by UDZ2 are largely comparable to the daily estimates obtained using top-quality geodetic receivers, UDT2 and UDI2 (Figure 3);

(ii) The Eastern component of the time series at UDZ2 appears as being shifted, by about 0.6–0.7 mm, with respect to the East component at UDI2 and UDT2; this can be also observed in the histograms of the differences (Figure 4b) and it suggests a minor accuracy in the estimated results of UDZ2 along the East direction if compared with the other components of the displacement;

(iii) The differences are very small for the horizontal components, with displacement estimates differences less than 1 mm between the two geodetic-class receivers, UDI2 and UDT2, and less than 2 mm between UDZ2 (the low-cost receiver) and the two geodetic class receivers (Table 2). In the vertical component the maximum differences have up to four times higher values than horizontal ones, ranging from about 5 mm between the estimations made by UDZ2 and those made by UDI2, and 8.3 mm between the two top-class receivers, UDI2 and UDT2. Hence, we can infer that the differences are in the order of some millimeters (1–2 on horizontal components and 5–8 mm on the vertical ones) in the estimated displacements are usual, and probably ascribed to the receiver brand or type, but this fact does not compromise the reliability of the results. The cost-effective u-blox F9P device used in our study reaches same-level estimates of top-quality geodetic instruments, in both horizontal and vertical components;

(iv) The cost-effective GNSS receiver u-blox F9P, in combination with the geodetic-class antenna, allows achieving mm-order precision results comparable with the precision of geodetic-class receivers; the best performance is achieved in the horizontal components, where the precision is sub-millimetric: the standard deviation (STD) is 0.2 mm for the differences between the two geodetic-class receivers and about 0.4 mm for the differences between the low-cost receiver and each of the two geodetic class; in the vertical component the precision achieved is minor instead, though std remains below 2.5 mm (Table 2); this result is in agreement with the previous studies, despite they consider real-time positioning results [5,7,9];

(v) If we consider the general trends of displacement, all the three stations of the MS are moving towards the North and upwards (Figure 3), and this horizontal motion is consistent with the regional tectonic trend in the area [19,20,21,22]. The linear trends values for each component at UDZ2 are very similar to those at UDI2 and UDT2, despite the errors being slightly higher, even remaining below 0.5 mm in the horizontal axes, and reaching 2 mm in the vertical. We do not discuss further the specific trend values obtained, since 5 months is a very short time period to estimate a reliable tectonic trend value and tectonic implications are out of the scope of this study.

## 4. Concluding Remarks

We tested a new system for crustal deformation purposes, composed of a geodetic-class antenna and three parallelly connected GNSS permanent receivers, two of which are top-class geodetic instruments, and one is a cost-effective dual frequency receiver. We processed the data along with the observations coming from a network of GNSS permanent stations active in NE-Italy. We analyze the data of five months, comparing the time-series, and the precisions and accuracies obtained.

Though some discrepancies in the performance of the three stations were expected, due to the difference in the receivers’ price, results are encouraging. On one hand, we observe that: (i) the daily estimates obtained by using the cost-effective u-blox F9P device are largely comparable to the daily estimates obtained using geodetic-class receivers, reaching sub-millimetric precision in the horizontal components and less than 2.5 mm in the vertical one; (ii) the general trends obtained by linearly fitting the time series are very similar to each other and show the motion of the sites towards the North and upwards, in agreement with the most recent studies on the tectonic deformation in the study area; and (iii) the analysis of the differences indicate that differences in the order of some millimeters (1–2 mm in the horizontal components and 5–8 mm in the vertical one) in the estimated displacements are usual, and probably ascribed to the receiver brand or type, but this fact does not compromise the reliability of the results. On the other hand, we notice that the accuracy in the displacement estimates is comparable with the geodetic class receivers for the vertical and northern components, and lower in the eastern component. Furthermore, we find a not negligible difference in the number of observations retrieved. The geodetic-class receivers show they are capable of retrieving more than the 90% of the observations, whereas the low-cost u-blox F9P device is capable of retrieving only 60–70% for most of the days, which is a worthwhile amount for a low-cost instrument, considering also the high precision it is able to achieve.

Despite these drawbacks, the capability of the u-blox F9P receiver of producing high-precision data, which are largely comparable to those of top-class geodetic instruments, makes this instrument a high-performing, cost-efficient, and reliable alternative, not only for monitoring purposes (landslides, ice sheets, etc.), but also for crustal deformation studies.

## Figures and Tables

**Table 1:** Main characteristics of the GNSS receivers used in this study.

Receiver Type	Indicative Price * (EUR)	Satellite Constellations Tracked	Carrier Frequency	GNSS Channels #	Maximum Sampling Rate
GNSS cost-effective device	~1000	GPS, GLONASS, GALILEO, Beidou, QZSS, SBAS	GPS: L1C/A, L2C GLONASS: L1OF, L2OF; GALILEO: E1B/C, E5b; Beidou: B1I, B2I; QZSS: L1C/A, L1S, L2; SBAS: L1C/A	184	20 Hz
Topcon TPS NETG5	~10,000	GPS, GLONASS, GALILEO, Beidou, SBAS, QZSS	GPS: L1 C/A, L1C, L1P(Y), L2P(Y), L2C, L5; GLONASS: L1 C/A, L1P, L2 C/A, L2P, L3C; GALILEO: GIOVE-A/B, E1b, E1, E5a, E5b, E6, AltBOC; Beidou: B1, B2, B3; SBAS: WAAS/EGNOS/MSAS; QZSS: L1 C/A, L1C, L2C, L5, LEX	452	100 Hz
Leica GR25	~10,000	GPS, GLONASS, GALILEO, Beidou, SBASS, QZSS, IRNSS	GPS: L1, L2P(Y), L2C, L5; GLONASS: L1, L2P, L2C, L3; GALILEO: E1, E5a, E5b, AltBOC, E6; Beidou: B1, B2, B3; SBAS: WAAS/EGNOS/MSAS/GAGAN; QZSS: L1, L2C, L5; IRNSS: L5	555	50 Hz

* Prices indicated in the table should be considered indicative of the order of magnitude of the real price. More detailed information should be asked to the authorized vendors. **#** in the number of available GNSS channels and in the sampling rate capability (further details can be found at https://kb.unavco.org/kb/article/unavco-resources-gnss-receivers-434.html, accessed on 1 December 2021).

**Table 2:** Differences in the daily displacements estimates between the station in the first column (site 1) and the station in the second column (Site 2). Last two columns indicate the mean and standard deviation of the differences.

Site 1 (Receiver Type)	Site 2 (Receiver Type)	Displacement Component	Maximum Difference Range (mm)	Mean (mm)	STD (mm)
UDT2 (TPS NETG5)	UDI2 (Leica GR25)	dN	0.74	−0.09	0.19
UDT2 (TPS NETG5)	UDI2 (Leica GR25)	dE	0.63	−0.09	0.21
UDT2 (TPS NETG5)	UDI2 (Leica GR25)	dU	8.28	0.25	1.76
UDZ2 (u-blox F9P)	UDI2 (Leica GR25)	dN	1.54	0.17	0.48
UDZ2 (u-blox F9P)	UDI2 (Leica GR25)	dE	1.81	−0.69	0.40
UDZ2 (u-blox F9P)	UDI2 (Leica GR25)	dU	5.02	0.29	1.92
UDZ2 (u-blox F9P)	UDT2 (TPS NETG5)	dN	1.76	0.25	0.49
UDZ2 (u-blox F9P)	UDT2 (TPS NETG5)	dE	1.89	−0.6	0.45
UDZ2 (u-blox F9P)	UDT2 (TPS NETG5)	dU	7.68	0.03	2.41

## Data Availability

Data used in the experiment are available on the public archive FReDNet (http://frednet.crs.inogs.it, accessed on 1 December 2021).
